# The Stress-Inducible Protein DRR1 Exerts Distinct Effects on Actin Dynamics

**DOI:** 10.3390/ijms19123993

**Published:** 2018-12-11

**Authors:** Anja Kretzschmar, Jan-Philip Schülke, Mercè Masana, Katharina Dürre, Marianne B. Müller, Andreas R. Bausch, Theo Rein

**Affiliations:** 1 Max Planck Institute of Psychiatry, Kraepelinstraße 2-10, 80805 München, Germany; anja.kretzschmar@mytum.de (A.K.); jan.schuelke@gmail.com (J.-P.S.); mmasana@ub.edu (M.M.); marianne.mueller@unimedizin-mainz.de (M.B.M.); 2 Department of Psychiatry and Psychotherapy & Focus Program Translational Neuroscience, Johannes Gutenberg Universität Medical Center, 55131 Mainz, Germany; 3 Department of Biomedical Sciences, Faculty of Medicine and Health Sciences, University of Barcelona, IDIBAPS, CIBERNED, Casanova, 143, 08036 Barcelona, Spain; 4 Lehrstuhl für Biophysik E27, Technische Universität München, 85748 Garching, Germany; katharina.duerre@tum.de (K.D.); abausch@mytum.de (A.R.B.)

**Keywords:** stress physiology, cytoskeleton, actin dynamics, DRR1, TU3A, FAM107A

## Abstract

Cytoskeletal dynamics are pivotal to memory, learning, and stress physiology, and thus psychiatric diseases. Downregulated in renal cell carcinoma 1 (DRR1) protein was characterized as the link between stress, actin dynamics, neuronal function, and cognition. To elucidate the underlying molecular mechanisms, we undertook a domain analysis of DRR1 and probed the effects on actin binding, polymerization, and bundling, as well as on actin-dependent cellular processes. Methods: DRR1 domains were cloned and expressed as recombinant proteins to perform in vitro analysis of actin dynamics (binding, bundling, polymerization, and nucleation). Cellular actin-dependent processes were analyzed in transfected HeLa cells with fluorescence recovery after photobleaching (FRAP) and confocal microscopy. Results: DRR1 features an actin binding site at each terminus, separated by a coiled coil domain. DRR1 enhances actin bundling, the cellular F-actin content, and serum response factor (SRF)-dependent transcription, while it diminishes actin filament elongation, cell spreading, and actin treadmilling. We also provide evidence for a nucleation effect of DRR1. Blocking of pointed end elongation by addition of profilin indicates DRR1 as a novel barbed end capping factor. Conclusions: DRR1 impacts actin dynamics in several ways with implications for cytoskeletal dynamics in stress physiology and pathophysiology.

## 1. Introduction

Stress is a risk factor for several pathologies, including mental disorders such as psychiatric diseases [1,2]. Underlying mental disorders are alterations in the pattern of synaptic structure and activity, which has been repeatedly shown to be impacted by stress [2,3]. Actin, as the most prominent cytoskeletal component at the synapse, plays a major role in synaptic transmission by regulating synaptic shape, neurotransmitter vesicle release, and post-synaptic receptor distribution [4]. Actin dynamics and rearrangements of actin filaments are crucial during structural and functional alterations of neurons in response to stress shaping synaptic plasticity and behavior [5]. More specifically, acute and chronic stress have been shown to dramatically impact on numerous processes, including neuronal architecture, network dynamics, synaptic efficacy, and dendritic spine shape [2,6]. Further, dynamics of dendritic spines have been implicated in both memory formation and the development of psychiatric or neurological disorders [7]. Dysregulation of synaptic actin dynamics has been proposed as a convergent mechanism of mental disorders [8]. Therefore, investigating how specific actin binding proteins modulate actin dynamics is essential to understanding cell physiology and disease pathophysiology. Since the actin cytoskeleton exerts a major modulatory function in a plethora of additional cellular processes such as morphogenesis, motility or endocytosis, deciphering the processes contributing to dynamic actin cytoskeleton rearrangements is relevant to understanding several human pathologies [9].

The variety of actin-dependent processes is accomplished by its highly dynamic structure: globular actin (G-actin) polymerizes to filamentous actin (F-actin), while this polymerization reaction and the organization of actin filaments to higher-order actin-structures is orchestrated by numerous actin binding proteins. Adenosine triphosphate (ATP)-bound actin monomers are added at the barbed (+) end of the filament, ATP is then hydrolyzed by actin along the filament leading to its destabilization and depolymerization at the opposite, pointed (−) end. Thereby, actin filaments undergo a constant turnover of monomers called treadmilling [10].

The rate-limiting step of filament polymerization, the formation of actin dimers and trimers, is enhanced by nucleating factors like formins [11,12]. In contrast, the Arp2/3 complex generates new actin filaments by nucleation from existing filaments [13]. Elongation is terminated by capping proteins that bind to the barbed ends and inhibit the addition of further actin monomers, thereby limiting the length of the filament [14,15].

While sheet-like structures necessary for lamellipodial protrusions of the cells are created by Arp2/3 and crosslinkers like filamin, finger-like filopodia are arranged by thick actin bundles crosslinked, e.g., by fascin or α-actinin [16,17,18]. The cellular G-/F-actin equilibrium further changes the intracellular processes, for example, the transcription factor serum response factor (SRF) [19]. SRF-responsive genes, in turn, encode regulators of the actin and microtubule cytoskeleton, cell growth, and motility, adhesion, extracellular matrix synthesis and processing, and transcription [20].

Previously, we have identified a novel stress-induced protein enhancing cognition and social behavior, primarily localizing to actin-rich structures like stress fibers, membrane ruffles, and synapses [21,22,23]. This protein had initially been described as a tumor suppressor and, thus, had been termed downregulated in renal cell carcinoma gene 1 (DRR1). It is also known as Tohoku University cDNA clone A on chromosome 3 (TU3A) or Family with sequence similarity 107, member A (FAM107A) [24,25]. DRR1 is downregulated in various cancer cell lines, including renal cell, ovarian, cervical, laryngeal, gastric, prostate, liver, lymph, and non-small cell lung cancer and is associated with the progression of neuroblastoma, meningioma and malignant glioma [26,27,28,29,30,31,32,33,34,35,36]. On the other hand, DRR1 is highly expressed in outer radial glial cells [37] and in the invasive component of glioblastoma [38,39]. Lately, DRR1 has been associated to several brain disorders. Gene expression analyses indicated altered expression of DRR1 in neurodegenerative diseases as well as in bipolar disorder, autism spectrum disorder, and schizophrenia, presumably indicating an aberrant adaptation to chronic stress [40,41,42,43,44,45,46].

DRR1 shows basal expression in several brain regions and is strongly upregulated in mouse models of stress, as well as by dexamethasone in the hippocampus [23,47,48,49]. Its virus-mediated upregulation—aiming at mimicking stress-induced DRR1 increase—in the Cornu Ammonis region 3 (CA3) hippocampal region and the lateral septum increased hippocampus-dependent memory and social behavior, respectively [22,23]. Recently, cognitive impairment was measured 4 h after social defeat stress, when DRR1 protein levels were not increased yet, but not after 8 h, when DRR1 protein levels were found increased [50]. However, viral-mediated overexpression of DRR1 was not able to prevent the cognitive impairments 4 h after social defeat [50]. These findings suggest DRR1 to act as an adaptation factor that contributes to the molecular machinery counterbalancing aversive stress effects, but cannot act in a preventive manner. On the molecular and cellular levels, it was found to directly interact with β-actin and inhibit neurite outgrowth [23].

The link between stress and actin dynamics appears to be a critical component of the general adaptation mechanism [5,22,23]. However, up to now, a more detailed mechanistic understanding of DRR1’s action on actin is lacking. Given the relevance of DRR1 not only during the stress response, but also in brain disorders and tumor development and progression, we aimed at elucidating its molecular mechanism and its significance in actin-dependent cell function. We found that DRR1 impacts actin dynamics in an intriguing multifaceted fashion by bundling, capping and nucleating filaments, altogether leading to stabilization of F-actin.

## 2. Results

### 2.1. DRR1 Features an Actin Binding Site at Each Terminus

**Figure 1:**
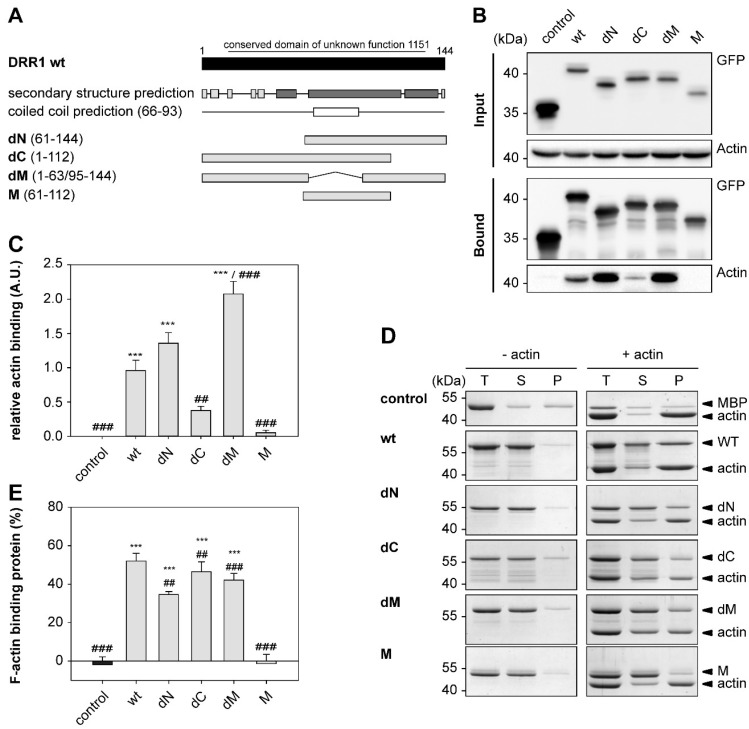
Downregulated in renal cell carcinoma 1 (DRR1) features an actin binding site at each terminus. (**A**) Domain structure of DRR1 wt and mutants. DRR1 harbors a conserved domain of unknown function from amino acid 16–133. Secondary structure prediction was performed with the “Predict Protein Server” (dark: helix, light: loop; https://www.predictprotein.org/, accessed on 31 July 2012). Coiled coil prediction performed with “Coils” (http://embnet.vital-it.ch/software/COILS_form.html; accessed no 10 December 2018); (**B**) Co-immunoprecipitation of actin with DRR1 wt and mutants fused to Enhanced Green Fluorescent Protein EGFP overexpressed in Human embryonic kidney 293 cells (HEK)-293 cells using Green Fluorescent Protein (GFP)-Trap^®^ beads. Control was performed with EGFP alone. Lysate and eluate samples were analyzed by SDS-PAGE and Western blot. A representative Western blot is shown; (**C**) Quantification of Co-immunoprecipitation (*n* = 8, dN and M *n* = 7); (**D**) Co-sedimentation of recombinant wt and mutant DRR1 protein with preformed F-actin by ultracentrifugation. Coomassie-stained sodium dodecyl sulfate (SDS) – polyacrylamide gel electrophoresis (PAGE) with total (T), supernatant (S) and pellet (P) fractions are shown; (**E**) Quantification of co-sedimented protein (*n* = 3). Bars represent means + SEM. **^/##^ *p* < 0.01, ***^/###^ *p* < 0.001 in comparison to control/wt DRR1 (only significant differences are marked). Statistical analysis was performed with one-way analysis of variants (ANOVA) and Bonferroni post hoc.

Murine DRR1 is a highly conserved protein with 144 amino acids containing the “conserved domain of unknown function 1151”. Secondary structure prediction in DRR1 indicates a predominantly helical protein with three helices and a coiled coil motif from amino acids 66 to 93 within the central helical region. Coiled coil motifs are abundant in the eukaryotic proteome and frequently involved in protein-protein and protein-DNA interactions [51,52]. Based on this structure prediction, DRR1 was divided into three domains: an N-terminal domain, a middle domain, and a C-terminal domain. Truncation mutants were generated accordingly to map the functions of DRR1 on actin dynamics in vitro and actin-dependent cellular processes (Figure 1A).

Actin binding of wild-type (wt) and mutant DRR1 was verified by co-immunoprecipitation (CoIP) from cellular extracts and by co-sedimentation of purified recombinant DRR1 proteins with F-actin. For immunoprecipitation, enhanced green fluorescent protein (EGFP)-tagged DRR1 proteins were ectopically expressed in Human embryonic kidney 293 cells (HEK)-293 cells. CoIP revealed actin binding of wt and all mutants except for the middle domain M. Quantification of relative actin binding in the CoIP revealed significant binding for DRR1 wt, dN, and dM, while it was not significant for dC and M. However, some actin binding could still be detected for dC in the Western blot. This is consistent with the presence of an actin binding site at both the N- and the C-terminus (Figure 1B,C).

For the co-sedimentation assays, purified G-actin from rabbit skeletal muscle was polymerized and then incubated with purified wt and mutant DRR1 proteins (tagged with maltose binding protein (MBP)) followed by high speed centrifugation and analysis of the total (T), supernatant (S), and pellet (P) fractions by Sodium dodecyl sulfate polyacrylamide gel electrophoresis (SDS-PAGE) and Coomassie staining (Figure 1D). Largely consistent with the results from the CoIP, there was significant binding to F-actin in the co-sedimentation assay for DRR1 wt and the mutants dN, dC and dM. The mutant M showed no binding (Figure 1D,E). In comparison to wt, deletion of the N, M and C domain modulated actin binding, which appeared more pronounced in the coprecipitation experiment (Figure 1 C,E), while deletion of both N and C domain completely abolished it.

### 2.2. DRR1 Enhances Actin Bundling Via Its Two Actin Binding Regions and Potentially through Homo-Dimerization

**Figure 2:**
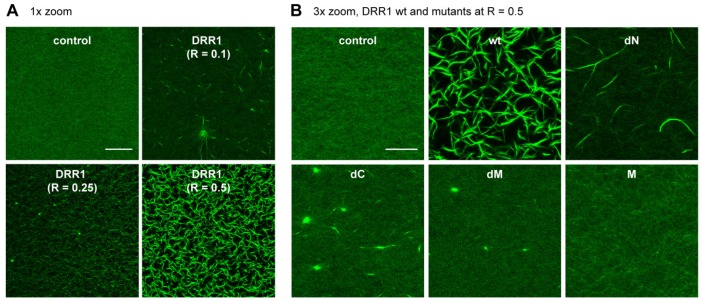
DRR1 enhances actin bundling via its two actin binding regions and potentially through homo-dimerization. (**A**) DRR1 enhances bundling of F-actin in a concentration-dependent manner. Z-stacks from actin networks polymerized at room temperature (RT) for >2 h in the presence of DRR1 and visualized with phalloidin-488. Scale bar denotes 50 µm. In all panels, “R” refers to the molar ratio of recombinant protein: actin protein; (**B**) Z-stacks from actin networks polymerized at RT for > 2 h in the presence of DRR1 proteins (R = 0.5) and visualized with phalloidin-488. Control = MBP added, because the DRR1 proteins are MBP-tagged. Scale bar denotes 20 µm.

To visualize the DRR1-induced alterations in F-actin networks, in vitro actin networks were polymerized in the presence or absence of recombinant DRR1 (purified via the MBP-tag) until equilibrium and then imaged in a confocal microscope. While the networks of the control (MBP added) showed a purely filamentous network lacking distinguishable higher order structures, the addition of DRR1 resulted in strong bundle formation with a completely bundled network in a concentration-dependent manner (Figure 2A), consistent with our previous results [23]. At a DRR1:actin ratio (R) of 0.1, DRR1 already generates clear actin bundling. At the highest DRR1:actin ratio tested of R = 0.5, the whole network appears as bundles without distinguishable single filaments.

To dissect the bundling mechanism of DRR1, actin networks polymerized in the presence of each mutant at R = 0.5 were also visualized in the confocal microscope (Figure 2B). Despite of retaining both actin binding regions, addition of the mutant dM only led to amorphous bundle “aggregates”, but no proper actin bundling. This suggests that the central region is necessary as a spacer for accurate positioning of the two actin binding regions for proper bundle formation. In contrast, the mutants dN and dC both impacted on actin networks by producing bundle-like structures, although they both harbor one actin binding region only. While dC showed bundle-like aggregates, dN generated actin bundling comparable to wt DRR1 at a lower concentration (compare Figure 2B dN R = 0.5 and Figure 2A wt R = 0.1). These findings could be explained by homo-dimerization of the mutants dC and dN through the putative coiled coil interaction motif (compare with Figure 1). The mutant M exhibited no effect on the actin networks and appeared similar to the control. This is consistent with the lack of F-actin binding observed in Figure 1.

**Figure A1:**
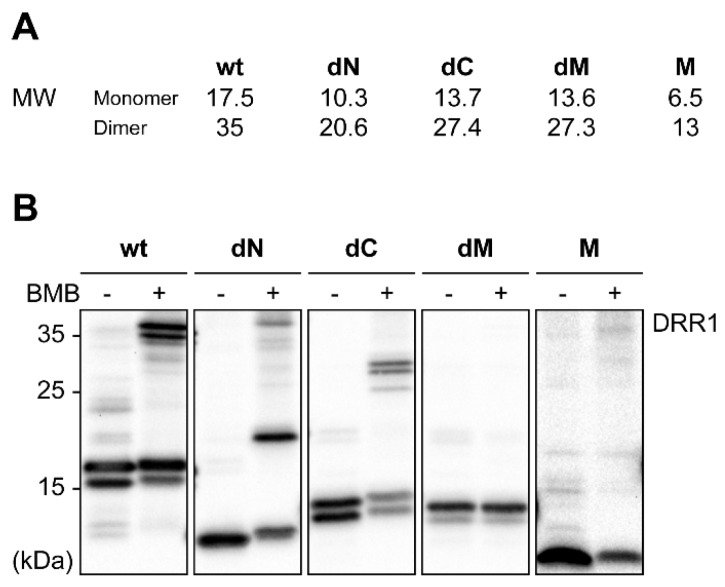
Crosslinking with BMB of DRR1 proteins from HEK-293 cell extracts suggests potential dimerization of DRR1 wt, dN and dC. (**A**) Expected molecular weight (MW) of DRR1 monomers and dimers in kDa; (**B**) HEK-293 cells transfected with wt or mutant DRR1 (no tag) were incubated with the cysteine-cysteine crosslinker BMB (+) or DMSO (−) as a control. Lysates were analyzed by SDS-PAGE and Western blot. Representative Western blots are shown.

In an effort to provide experimental evidence for the dimerization of DRR1, we expressed untagged wt and mutant DRR1 in HEK293 cells and probed for dimerization using the crosslinker 1,4-Bismaleimidobutane (BMB) that reacts with sulfhydryl groups (cysteines) in close proximity. Western blot analysis revealed signals at the dimer positions of wt DRR1, dC and dN, but not for dM and M (Figure A1). Thus, as predicted by the secondary structure analysis (Figure 1), DRR1 dimerizes very likely through the middle domain that features the only cysteine (position 94) available for crosslinking by BMB. However, it appears that actin binding strongly promotes dimerization, because the M-domain alone did not produce any sign of dimerization, probably because it is too dispersed throughout the cell. It also should be noted that on formal grounds, these data do not exclude the possibility that the N- and the C-domain each dimerize independently.

### 2.3. DRR1 Bundling Diminishes Cellular Actin Treadmilling

**Figure 3:**
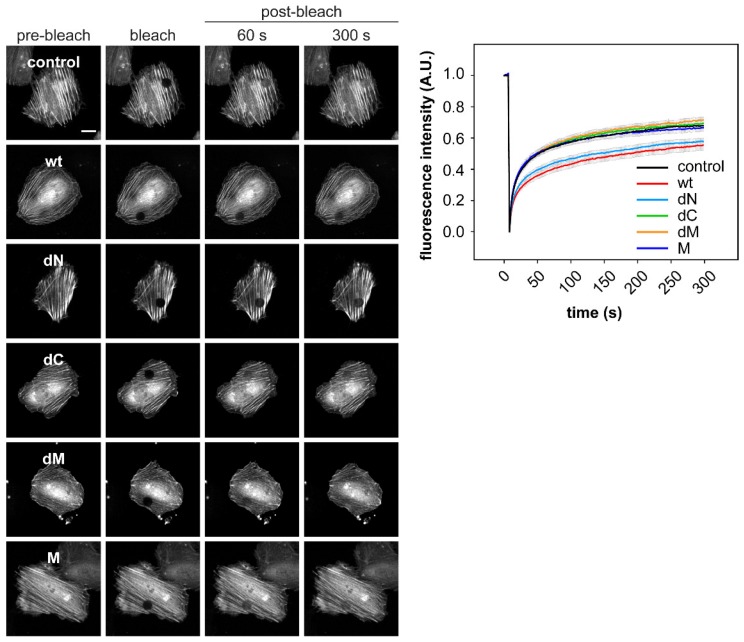
DRR1 bundling effect diminishes cellular actin treadmilling. DRR1 wt and the mutant dN—but none of the other mutants–slow down actin treadmilling in HeLa cells. Fluorescence recovery after photobleaching (FRAP) in HeLa cells co-transfected with plasmids expressing GFP-actin and untagged DRR1 wt, dN, dC, dM, and M was recorded. Representative cells are shown. Quantification was performed in ImageJ (25–30 cells from 2–3 independent experiments). Scale bar denotes 20 µm. Movies of FRAP experiments are available on request.

The strong bundling effect of DRR1 on actin filaments in vitro could lead to a stabilization of F-actin as well as reduced actin treadmilling in cells. To test this hypothesis, fluorescence recovery after photobleaching (FRAP) of Green Fluorescent Protein (GFP)-labeled actin was measured in HeLa cells co-expressing untagged DRR1 (or empty vector as control) for 24 h. Time-lapse images were acquired with a confocal microscope during 5 min (five frames were recorded pre-bleach). After 5 min, the recovery of fluorescence in control cells reached about 75% of the bleached fluorescence. In DRR1 wt overexpressing cells, however, the recovery reached about 55%, indicating a higher immobile fraction of actin and, thus, a reduced actin treadmilling rate. None of the deletion mutants analyzed changed FRAP, except for the mutant dN, which exerted a similar effect as wt DRR1 (Figure 3).

**Figure A2:**
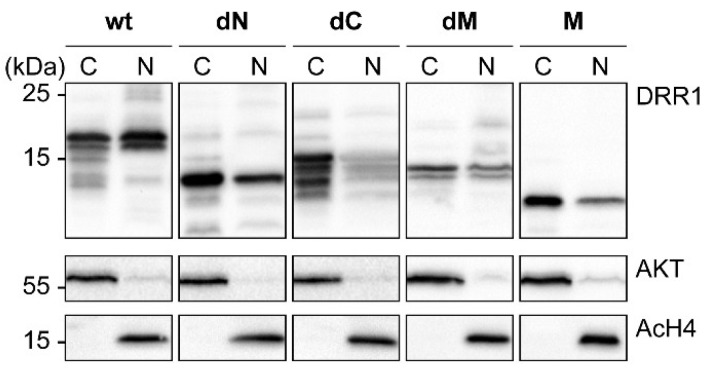
DRR1 wt and mutants display nuclear localization. Cytosolic and nuclear fractions of HEK-293 cells expressing ectopic DRR1 wt or mutants were analyzed by SDS-PAGE and Western blot. Efficiency of fractionation into cytosol (C) and nucleus (N) was confirmed with antibodies against the cytosolic kinase AKT and the nuclear acetyl-histone H4. Representative Western blots are shown.

The images of Figure 3 display nuclear localization of Actin, consistent with other reports [53]; DRR1 also has been reported to be both in the nuclear and cytosolic compartment [25,47,54]. We performed biochemical fractionation of HEK-293 cells ectopically expressing wt or mutant DRR1 to analyze the nuclear and cytosolic fraction by Western blotting (Figure A2). The efficiency of fractionation was monitored by probing the membranes for the cytosolic kinase AKT and the nuclear histone H4 (acetylated). All DRR1 forms could be detected both in the cytosol and in the nucleus (Figure A2).

### 2.4. DRR1 Reduces Actin Filament Elongation but Increases Nucleation

**Figure 4:**
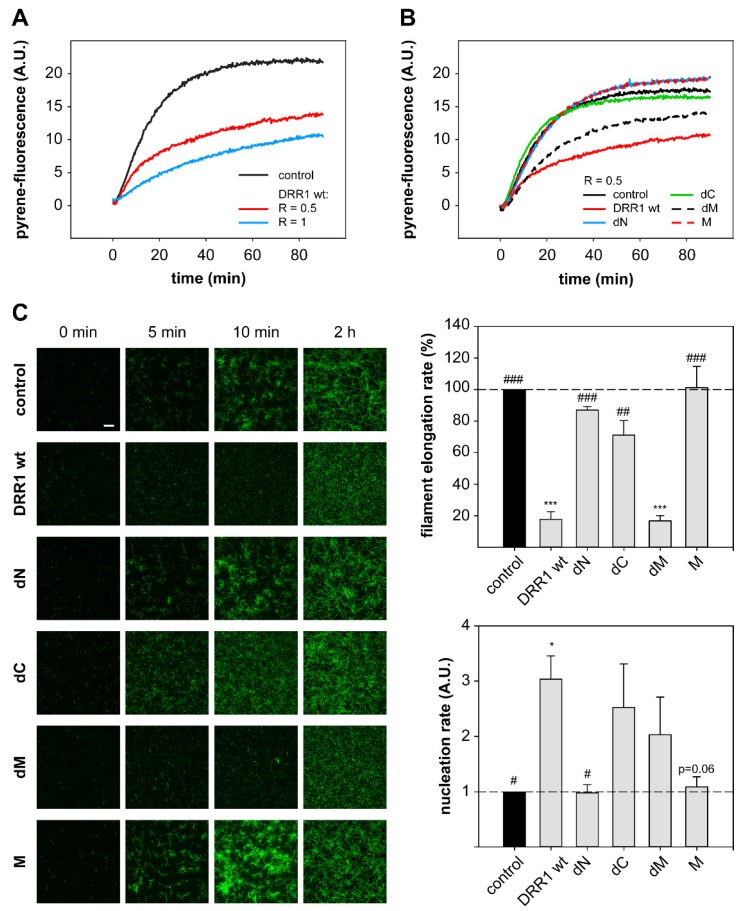
DRR1 reduces actin filament elongation but increases nucleation. (**A**,**B**) DRR1 and the mutant dM exert an inhibitory effect on in vitro polymerization of pyrene-actin. 20% pyrene-labeled actin (4 µM) was polymerized in the presence of wt (**A**,**B**) and mutant (**B**) DRR1 proteins (purified via the MBP-tag) as indicated. Increase in fluorescence of pyrene-actin during polymerization was monitored in 5 s intervals for 90 min; (**C**) Single filament elongation of actin is strongly reduced by DRR1 and the mutant dM. Actin (c = 0.5 µM, 10% labeled with ATTO-488) was polymerized in the presence of DRR1 proteins or MBP as control (R = 0.5) and visualized by TIRF microscopy for 10 min with 3 s intervals starting 2 min after the beginning of the reaction. An endpoint image was taken at 2 h of polymerization. Scale bar denotes 10 µm for all images. Bars indicating the filament elongation rate and the nucleation rate represent means + SEM of three independent experiments. */^#^ *p* < 0.05, **/^##^ *p* < 0.01, ***/^###^ *p* < 0.001 in comparison to control/wt DRR1 (only significant differences are marked; *p* = 0.06 refers to the comparison of M to wt DRR1). Statistical analysis was performed with one-way ANOVA and Bonferroni post hoc. Movies of single filament elongation experiments are available on request.

To explore the effects of DRR1 on actin beyond the previously described bundling ability of DRR1 [23], we examined actin polymerization with pyrene-actin in the presence of DRR1 (Figure 4A,B) as well as single filament elongation and nucleation using total internal reflection fluorescence (TIRF) microscopy (Figure 4C).

As a first step to analyze actin polymerization in the presence of DRR1, pyrene-labeled actin, which shows enhanced fluorescence upon polymerization, was polymerized in the presence of increasing concentrations of DRR1 wt. At a DRR1:actin ratio of R = 0.5 and 1, the polymerization reaction was strongly inhibited in comparison to the control (Figure 4A). The only mutant to have an effect comparable to DRR1 was dM (Figure 4B).

In order to verify the slowdown of polymerization by DRR1, the polymerization reaction of fluorescently labeled G-actin was monitored using TIRF. The overall slowdown of actin polymerization by DRR1 observed in the pyrene-assay was well reproduced in the TIRF polymerization. DRR1 significantly slowed down actin polymerization to less than 20% control (Figure 4C). The mutant dM was the only mutant to retain the inhibitory effect of wt DRR1 on filament elongation, even though it had previously not shown an effect on bundling. The mutant dC showed a mild reduction of filament elongation, which was not significant. This finding indicates that the reduction of single filament elongation by DRR1 is independent of actin bundling, but both actin binding sites are necessary to affect elongation, since mutants lacking one or both actin binding sites displayed no significant effect.

Intriguingly, the visualization of single filament elongation revealed more but shorter filaments in the presence of DRR1 versus the control. Thus, the number of new filaments per time frame was quantified and the slope of the resulting plot was determined as read-out of the nucleation rate. DRR1 moderately enhanced the filament nucleation rate up to three-fold above the control at a molar ratio of DRR1:actin of 0.5. The mutants dC and dM both showed a trend towards increased nucleation versus the control, although not statistically significant (Figure 4C).

### 2.5. In the Presence of Profilin, DRR1, and the Mutants dM and dC Block Elongation More Effectively, Suggesting DRR1 as a Novel Barbed End Capping Factor

Different actin binding factors interact in the cell to control overall actin dynamics. To reconstitute more complex conditions in vitro, we analyzed the effects of wt DRR1 and mutants on actin polymerization in the presence of profilin, a well-described blocker of pointed end polymerization. Thus, we assessed elongation exclusively from the barbed end.

**Figure 5:**
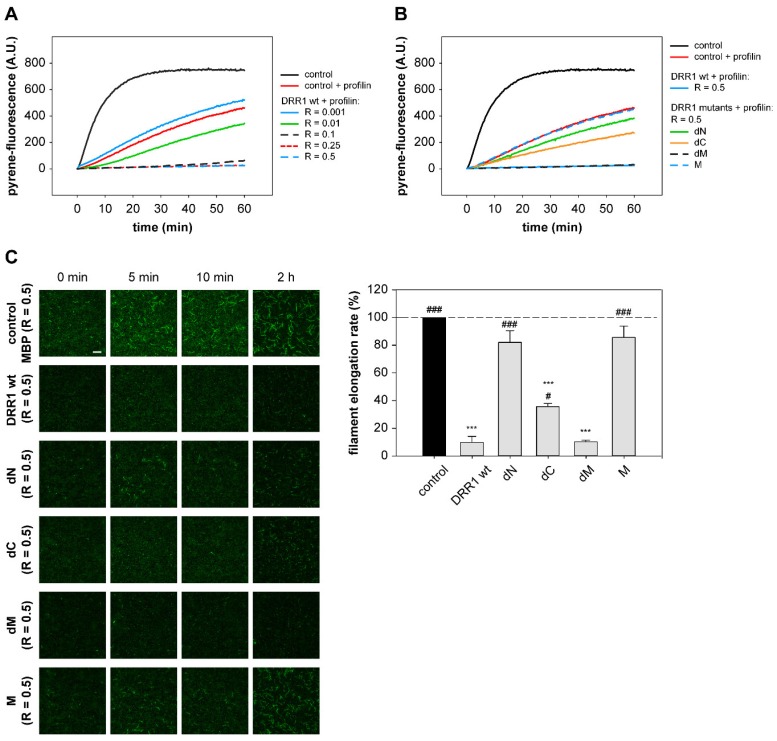
In the presence of profilin, DRR1 and the mutants dM and dC block elongation more effectively, suggesting DRR1 as a novel barbed end capping factor. (**A**,**B**) Pyrene-actin polymerization is blocked by DRR1 and the mutant dM at R = 0.5 in the presence of profilin (12 µM). 20% pyrene-labeled actin (4 µM) was polymerized in the presence of wt (**A**,**B**) and mutant (**B**) DRR1 proteins (purified via the MBP tag) as indicated. An increase in fluorescence of pyrene-actin during polymerization was monitored in 5 s intervals for 60 min; (**C**) Visualization of actin in vitro polymerization by TIRF microscopy (c = 0.5 µM, 10% labeled with ATTO-488) in the presence of profilin (1.5 µM). Actin was polymerized in the presence of DRR1 proteins for 10 min with 3 s intervals imaging starting 2 min after the beginning of the reaction. An endpoint image was taken at 2 h of polymerization. Scale bar denotes 10 µm for all images. Bars indicating the filament elongation rate represent means + SEM of three independent experiments. */^#^ *p* < 0.05, ***/^###^ *p* < 0.001 in comparison to control/wt DRR1 (only significant differences are marked). Statistical analysis was performed with one-way ANOVA and Bonferroni post hoc. Movies of single filament elongation experiments are available on request.

Addition of profilin strongly enhanced DRR1’s inhibitory effect on actin polymerization supporting the notion of DRR1 as a novel capping protein at the barbed end. In the pyrene-assay, an inhibitory effect of DRR1 on barbed end polymerization was already detectable at a DRR1:actin ratio of R = 0.01. At R = 0.1, polymerization was almost completely blocked (Figure 5A). At R = 0.5, the mutant dM most noticeably reduced actin polymerization, but also dC slowed down the polymerization, while all other mutants lacked strong effects (Figure 5B).

Similar results were obtained with polymerization of actin and single filament analysis using TIRF microscopy. At R = 0.5, DRR1 wt displayed a pronounced barbed end capping activity in the presence of profilin by reducing the filament elongation rate to about 10% of the control. The same effect was reproduced for dM. In addition, dC which had only a mild effect in the absence of profilin, reached significant capping activity in its presence reducing the filament elongation rate to around 36% of the control. Nevertheless, this capping activity of dC was less pronounced than for DRR1 wt (Figure 5C).

### 2.6. DRR1 Modulates Actin-Dependent Processes in Cells

To further analyze the cellular consequences of DRR1-induced changes of actin dynamics, we evaluated known actin-dependent processes such as cell spreading and activity of the transcription factor serum response factor (SRF). Cell spreading is relevant in many cellular functions, such as migration or wound healing. Spreading of HeLa cells ectopically expressing EGFP-DRR1 wt or mutants, was analyzed by replating on a fibronectin-coated surface and fixation after 30 min of spreading; F-actin was stained with phalloidin.

**Figure 6:**
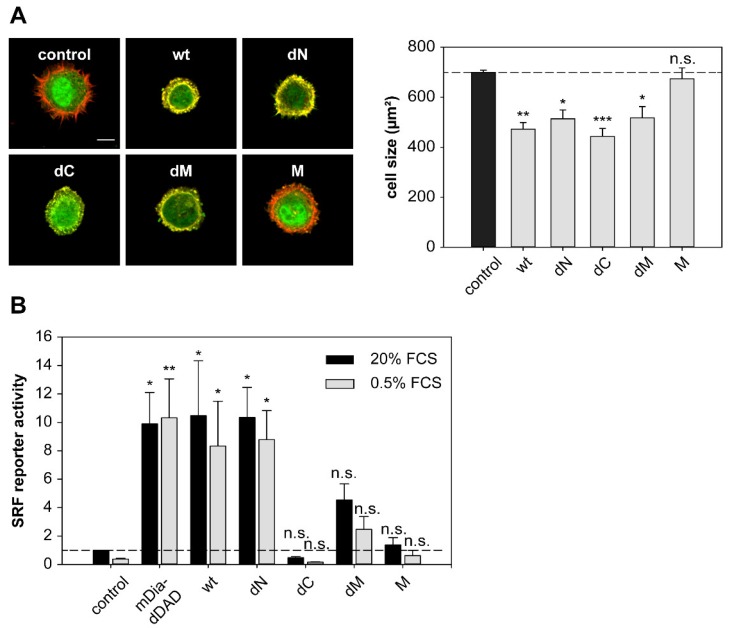
DRR1 modulates actin-dependent processes in cells. (**A**) DRR1 wt and the mutants dN, dC, and dM inhibit spreading of HeLa cells. Cells were transfected with constructs expressing EGFP-DRR1 wt or mutants (control: EGFP), cultivated for 24 h and re-plated on fibronectin-coated coverslips. After 30 min, cells were fixed and F-actin was stained with phalloidin. Representative cells are displayed (green: EGFP or EGFP-DRR1; red: F-actin). Scale bar denotes 20 µm. Bars represent mean cell sizes + SEM of four independent experiments (50–200 cells in each experiment). * *p* < 0.05, ** *p* < 0.01, *** *p* < 0.001 in comparison to control. Statistical analysis was performed with one-way ANOVA and Bonferroni post hoc; (**B**) DRR1 overexpression leads to a strong activation of the serum response factor (SRF) independently of serum, indicating a stabilization of cellular F-actin by DRR1 bundling and capping effects. SRF reporter gene assays in HEK-293 cells show 8–10 fold enhanced SRF activity after overexpression of DRR1 wt or dN with and without serum. Cells were transfected with the SRF reporter 3DA.luc, the gaussia luciferase control vector and the indicated plasmids or vector control. Serum stimulation or withdrawal was for 16–20 h. Luciferase activity is shown as the fold-increase of serum-stimulation over control samples. Bars represent means + SEM of five independent experiments. * *p* < 0.05, ** *p* < 0.01, n.s. = not significant in comparison to control. Statistical analysis was performed with one-way ANOVA and Bonferroni post hoc.

DRR1 wt strongly reduced spreading of HeLa cells: while (EGFP transfected) control cells showed a mean size of about 700 μm², DRR1 wt expressing cells had a mean cell size below 500 μm² (Figure 6A). In addition, control cells expressing EGFP showed extension of filopodial protrusions after 30 min of spreading, while DRR1 wt-expressing cells were still round–shaped, lacking any protrusions. In these freshly-seeded cells, DRR1 wt colocalized with F-actin at the cortex area of the cells, where the filaments’ barbed ends are oriented [55]; this is consistent with DRR1’s capping activity at the barbed ends, thereby inhibiting extension of protrusions during cell spreading. Evaluation of the deletion mutants of DRR1 revealed that all mutants except M also inhibited cell spreading. This indicates that either capping or bundling by DRR1 is sufficient to reduce cell spreading.

**Figure A3:**
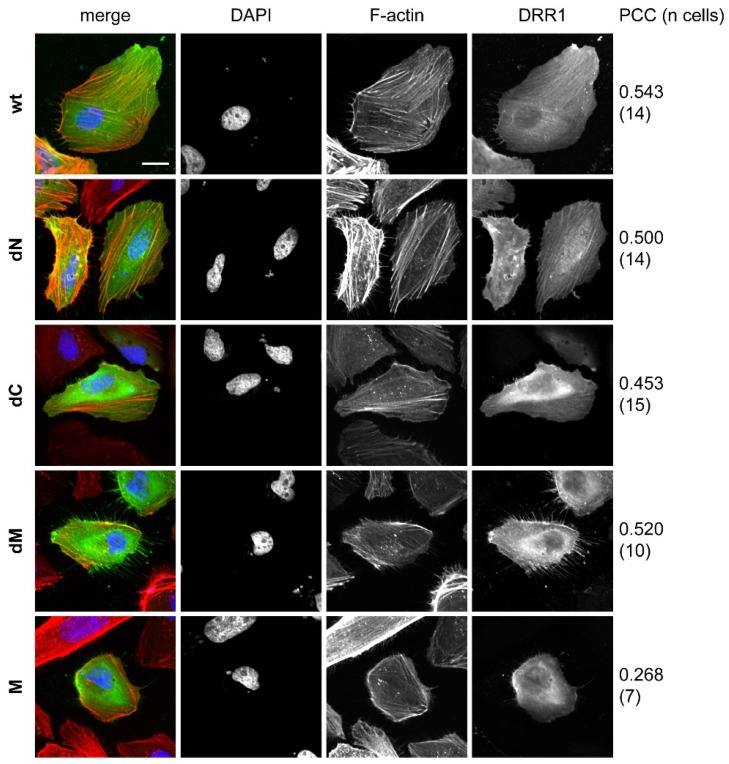
DRR1 wt and the mutants dN, dC and dM co-localize with F-actin in HeLa cells, particularly along stress fibers. HeLa cells were transfected with DRR1 plasmids or vector control and fixed 24 h after transfection. Nuclei were counterstained with DAPI (blue), F-actin with phalloidin (red) and DRR1 with a specific antibody (green). Representative cells are shown. Scale bar denotes 20 µm. Pearson’s correlation coefficient (PCC) and the number of cells used for colocalization analysis (n, from two independent experiments) are shown. DRR1 wt and the mutants dN, dC, and dM exhibit colocalization with PCCs above 0.4, which generally can be regarded as a strong positive correlation.

**Figure A4:**
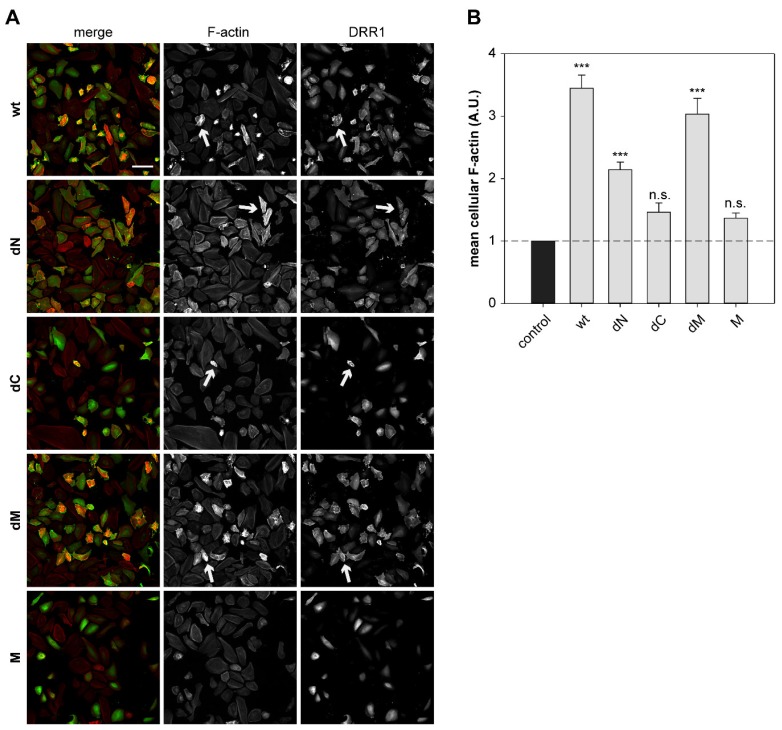
DRR1 modulates F-actin content in cultured cells. (**A**) HeLa cells were transfected with DRR1 plasmids and fixed 24 h after transfection. F-actin was stained with phalloidin (red) and DRR1 with a specific antibody (green). Scale bar denotes 100 μm. White arrows indicate exemplary cells with increased F-actin correlating with high DRR1 wt or mutant expression. (**B**) Mean F-actin per cell is increased upon overexpression of DRR1 wt and the mutants dN and dM. Quantification of mean cellular F-actin from HeLa cells described in A was performed and values of transfected cells were normalized to untransfected cells in the same image (number of cells in one experiment, control *n* = 200, DRR1 wt *n* = 102, dN *n* = 93, dC *n* = 38, dM *n* = 54, M *n* = 52). Bars represent means + SEM of five independent experiments. ** *p* < 0.01 in comparison to control. Statistical analysis was performed with one-way ANOVA and Bonferroni post hoc.

Cell imaging revealed that DRR1 colocalizes with F-actin (Figure 3 and [23]). As expected from the binding analyses (Figure 1), the mutants dN, dC, and dM also exhibit colocalization with actin filaments (Figure A3). Among them, dC, whose actin binding did not reach significance in the co-immunoprecipitation experiment (Figure 1C), exhibited the lowest correlation coefficient. M showed no colocalisation with F-actin (Figure A3). The overall cellular content of F-actin was increased by wt DRR1, dN, and dM, while dC and M had no significant effect (Figure A4).

The equilibrium between G- and F-actin has further repercussions for intracellular processes, for example activation of the transcription factor serum response factor (SRF). With decreasing levels of G-actin, the SRF cofactor MAL detaches from G-actin, translocates to the nucleus and activates SRF [19]. We employed SRF reporter gene assays as a G-actin sensor to monitor the effects of wt and mutant DRR1. The nucleator formin mDia lacking its autoinhibitory “DAD” region was used as a positive control for SRF activation [56].

DRR1 wt increased SRF activity about 10-fold in serum-stimulated cells, similar to the effect of mDia. In the absence of serum, the stimulation was still about eight-fold above the serum-stimulated control sample and again comparable to mDia, indicating strong SRF activation by DRR1 independently of serum. The mutant dN also significantly enhanced SRF activity, while the mutants dC, and M showed no effect. While dM showed a minor increase in SRF activity, its stimulation did not reach significance (Figure 6B). This data indicate that DRR1 expression levels modulate SRF-dependent gene expression through modulation of the equilibrium between G- and F-actin

## 3. Discussion

Actin binding proteins orchestrate the temporal and spatial remodeling of the actin cytoskeleton in cells as the structural basis for several cellular functions [57]. This highly dynamic process also responds to specific stimuli and, thus, conveys the ability to adapt to new environmental demands. Here, we present a domain and functional analysis of the stress-induced protein DRR1 with respect to its action on actin dynamics.

Our findings extend the characterization of DRR1 as actin bundler [23] and add it to the list of actin cappers. While the contact points of DRR1 on actin filaments remain unknown, capping might be achieved in two ways by the binding of at least one of the two actin binding domains of DRR1 described here close to the barbed end of the filament: either by inducing a conformational change at the outmost actin unit or by sterically interfering with the addition of the next actin molecule to the extending filament. Deletion of the N-terminal domain leads to complete loss of capping activity, while deletion of the C-terminus retains a somewhat lower capping activity. Therefore, we hypothesize that the N-terminal binding domain of DRR1 is required for capping. In addition, the second actin binding domain appears to contribute to capping possibly by stabilizing the interaction with actin. According to our model, the C-terminal deletion mutant dC is able to form dimers yielding two actin binding sites and, thus, enhances binding affinity to actin. Thus, the deletion mutant dC exerts some capping activity.

Most capping proteins appear to exert their activity at nanomolar concentrations [58], similar to the concentrations used here for DRR1. The ratio of DRR1 to actin of 1:10 at which significant capping was observed is also close to the range of other capping proteins, for example gelsolin [59]. However, the dominant actin capper in the cell, called “capping protein”, displays a very high binding affinity and is effective at ratios as low as 1:1000 [60]. Thus, the other capping proteins, like DRR1 and gelsolin, may have more specialized roles. In general, both actin interaction domains and capping mechanisms are not conserved. Nevertheless, the mode of action of DRR1 in capping at the barbed end might be similar to the mechanism proposed for Twinfilin [61,62] and the Gelsolin protein family [63,64,65]. These proteins feature multiple actin binding sites and contact the actin filament both at the barbed end and at the side of the filament.

We cannot exclude that the nucleation effect of DRR1 observed here in the in vitro assays could be secondary to the capping activity, i.e., due to the extended availability of non-polymerized actin when polymerization is diminished. For capping protein, a concentration-dependent nucleation activity has been reported: it inhibits elongation of actin already at low concentration by blocking the barbed end, while at higher concentrations it enhances nucleation by mimicking a non-dissociable actin dimer [14]. At this stage, it is likely that DRR1 may act as a nucleation factor by pulling together actin monomers or by stabilizing short oligomers, which appears possible in particular with dimerized DRR1 (compare also graphical abstract).

Efficient bundling of actin by DRR1 requires both binding sites of DRR1 as deletion of one of the domains severely compromises actin bundling. Reduced bundling was also observed for the deletion of the middle domain, suggesting that proper spacing of the two actin binding domains is required, possibly in conjunction with dimerization of the full length protein. Similarly, the residual bundling activity of each of the terminal deletion mutants might be attributed to their dimerization. Even though the middle domain does not bind to actin, our data do not allow excluding the possibility that it actively contributes to bundling.

Visually, with respect to bundle thickness and length, and mesh size of the bundled network, bundled actin networks with DRR1 and α-actinin, respectively, look similar. Furthermore, actin:DRR1 networks at a ratio of 1:2 are comparable to α-actinin:actin networks of 1:1 ([66] and this work), suggesting DRR1’s bundling activity to be at least as strong as the respective effect of α-actinin. Other actin bundling proteins inhibit in vitro actin depolymerization similarly to DRR1 in this study [67], and DRR1 itself has been shown to reduce dilution-induced actin depolymerization at ratios of DRR1:actin of 0.7 [23].

It should be noted that all experiments with recombinant DRR1 in this study had to be performed with a large (MBP) tag at the wt and mutant DRR1 proteins. Even though the cellular effects of DRR1 with a smaller (GFP) tag or with no tag reflect the in vitro results, and even though we distinguish the DRR1 effects from the effects of MBP alone, we cannot exclude the possibility that he MBP tag influenced the experimental outcome.

In cells, actin dynamics is shaped by the concerted action of several actin binding proteins. Although the effects observed in vitro with purified compounds may not always reliably predict the outcome in the cell, the effects on actin dynamics found in cells expressing wt DRR1 and its mutants were largely congruent with the in vitro results (summaries in Table 1 and Table 2). For example, the mutants that exerted proper bundle formation, i.e., DRR1 wt and dN, were the only ones to reduce actin treadmilling in the FRAP experiment, suggesting that it is mainly the bundling activity that leads to stabilization of F-actin in cells. We noted though, that the effect of dN in the FRAP assay is comparable to that of DRR1 wt, while the effect on bundling is not as strong (Figure 2). Thus, we cannot exclude the possibility that additional actions of dN contribute to the overall effect in the FRAP assay.

Both bundling and capping effects appear to contribute to activation of cellular SRF, since serum-independent SRF activation was observed for DRR1, dN, and dM. Meanwhile, bundling and inhibition of filament polymerization seem to be largely independent effects: dN generated bundles but had no effect on filament elongation, whereas dM had formed no proper bundles, but strong inhibition of filament elongation similar to the wild-type.

In general, cell spreading is known to be a complex process influenced by several parameters, including substrate stiffness and density and actin polymerization [68,69,70]. Early spreading was proposed to depend on the mechanical properties of the cell, and the actin cortex in particular [71]. In the cell spreading assay, mutants that showed bundling or capping activity (or both) exhibited an inhibitory effect. Presumably, bundling by DRR1 inhibits the early phases of spreading by increasing cell stiffness while capping likely interferes with extension of the lamella at the later stages of spreading. These results might explain the reduced spine density found in hippocampal neurons overexpressing DRR1 in rodents, and with the reduced neurite growth found in cultured Neuro2a cells [23]. The here-observed effect on cell spreading is unlikely to be a result of the function of DRR1 as tumor suppressor. Upon ectopic overexpression, no effect on cell viability or induction of apoptosis could be observed [23]. Furthermore, in the cell spreading assay only newly attached cells are followed 30 min after seeding. Conversely, we cannot exclude that impairment of cell spreading contributes to the tumor suppressive action of DRR1. However, another tumor suppressor, p14ARF, has been demonstrated to enhance cell spreading, reflecting its dual role in tumor suppression and apoptosis protection [72].

The recovery rate of GFP-fluorescence upon photobleaching reflects the actin turnover or treadmilling rate, as the free diffusion of monomeric G-actin is much faster [73,74,75]. It was somewhat surprising that the mutant lacking the middle domain, which displayed significant capping activity in vitro, did not affect actin turnover. Of the tested mutants only full length and the mutant dN showed an effect. It is, thus, possible that bundling of actin is causing the decrease in actin turnover. This was observed also for the actin bundling protein Ca^2+^/calmodulin-dependent protein kinase IIb (CaMKIIb), which reduced actin turnover in dendrites but did not directly impact on polymerization and depolymerization kinetics of actin [76]. Since actin treadmilling was shown to consume about half of the ATP pool in neurons [77], one of DRR1’s physiological roles upon stress could be saving ATP that might be required for the proper adaptive reaction to stress.

Increased levels of G-actin not only impact SRF, but have also been reported to reduce glucocorticoid receptor (GR)-dependent transcription, possibly through inducing the GR inhibitor c-jun [78]. Accordingly, the F-actin depolymerization factor cofilin 1 has been found to inhibit GR activity. Thus, it is possible that DRR1 enhances GR activity under certain conditions (constituting a feed-forward mechanism), which may furthermore be cell type-dependent because DRR1 is not only expressed in neurons, but also in other cell types, such as glial cells and various tissues [37,41,79].

Several studies proposed a role of actin dynamics and remodeling in psychiatric disorders such as depression, based on case-control comparisons and animal models [80,81,82,83]. A recent proteome study revealed increased levels of F-actin-capping protein subunit beta (CAPZB) in platelets from patients suffering from major depression in comparison to healthy controls [84]. Other studies reported changes of actin regulatory proteins by antidepressants and mood stabilizers [85,86] mutations in genes of the regulatory network of the actin cytoskeleton appear to be enriched in treatment-resistant major depression [87]. Pathway-based methods to genetic data have been suggested to blend biological information with the power of –omics approaches [83]; we propose that the stress- and glucocorticoid-regulated DRR1 [22,23,47,88] should be included when analyzing the role of the actin cytoskeleton in physiology and pathology, particularly in stress-related processes. Furthermore, since actin regulatory factors work in concert, future biochemical investigation of DRR1 should include the combination with additional actin binding proteins, as this study now firmly established DRR1 as an actin-regulatory protein.

## 4. Materials and Methods

Several of the methods outlined in the following are also described in the Ph.D. thesis of Anja Kretzschmar [89].

### 4.1. Plasmids

Plasmids for transfection in cell culture were cloned downstream of the Cytomegalovirus (CMV) promoter of the vector pRK5-SV40-MCS. DRR1 mutants were generated by PCR mutagenesis from murine DRR1 wild-type (wt) construct in pRK5. Cloning of the murine DRR1 wt construct was previously described in [23]. The nucleotide sequences of all constructs were confirmed after cloning by Sanger sequencing. For expression of DRR1 proteins N-terminally fused to EGFP or MBP, inserts of DRR1 wt and mutants were subcloned into the vector pEGFP-C1 (Clontech, Saint-Germain-en-Laye, France) or pMAL-CR1 (New England Biolabs, Ipswich, MA, USA), respectively. Details of the cloning strategies and primer sequences are available on request.

### 4.2. Cell Culture and Transfection

HeLa and HEK-293 cells were cultured in Dulbecco’s Modified Eagle Medium (DMEM, Life Technologies, Carlsbad, CA, USA) containing 10% fetal bovine serum, 1% sodium pyruvate, and 100 U/mL penicillin and streptomycin at 37 °C in a 5% CO_2_ atmosphere. A confluent 10 cm dish of HEK-293 cells was transfected by electroporation with 15 µg plasmid and cultured for two days until conduction of the experiments. HeLa cells were transfected using TurboFect (Thermo Scientific, Waltham, MA, USA) according to the manufacturer’s instructions and incubated for 24 h after transfection.

### 4.3. SDS-PAGE, Colloidal Coomassie Staining, and Immunoblot

Samples were separated on 10, 12, or 15% poly-acrylamide gels with 3.2% stacking gels and stained with colloidal coomassie brilliant blue G (Sigma-Aldrich, St. Louis, MO, USA) or electrophoretically transferred onto nitrocellulose membranes (GE Healthcare, Chalfont St Giles, UK). Immunodetection was performed by blocking the membrane with 5% non-fat milk in Tris-buffered saline, supplemented with 0.05% Tween (TBS-T, Sigma-Aldrich) for 1 h at room temperature, and then incubated with primary antibody overnight at 4 °C. The blots were washed and probed with the respective horseradish peroxidase- or fluorophor-conjugated secondary antibody for 3 h at room temperature. All antibodies were diluted in TBS-T with 2% milk powder. The immunoreaction was visualized with ECL detection reagent (Millipore, Darmstadt, Germany) or by fluorescence. The following antibodies were used: rabbit-anti-DRR1 (1:2000, Biogenes, Berlin, Germany, as described in [23]), goat-anti-actin (I-19, 1:2000, Santa Cruz Biotechnology, Dallas, TX, USA), mouse-anti-GFP (B-2, 1:2000, Santa Cruz Biotechnology), rabbit-anti-AKT (1:1000, Cell Signaling, Frankfurt, Germany), rabbit-acetyl-H4 (1:4000, Upstate, Schwalbach, Germany), donkey-anti-rabbit-HRP (1:10,000, Cell Signaling, Cambridge, UK), donkey-anti-goat-HRP (1:10,000, Santa Cruz, Heidelberg, Germany), and Alexa Fluor 488-donkey-anti-mouse (1:5000, Life Technologies). Determination of the relative optical density and quantification of band intensities were performed using the ImageLab 4.1 Software (Bio-Rad, Munich, Germany).

### 4.4. Protein Expression and Purification

**Figure A5:**
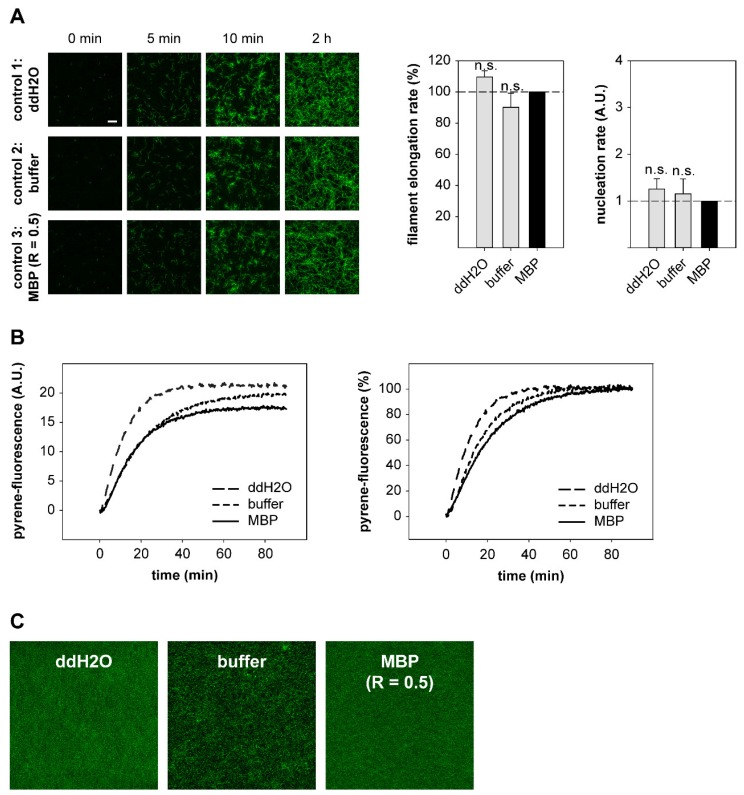
Overview of the controls used in all in vitro actin assays: ddH_2_O, buffer, and purified MBP protein. In all controls ddH_2_O and buffer were added in a constant volume, and MBP was added at the same ratio as DRR1 at the highest concentration, i.e., at R = MBP:actin = 0.5. (**A**) In vitro polymerization of actin visualized by TIRF microscopy for 10 min with 3 s intervals starting 2 min after the beginning of the reaction. An endpoint image was taken at 2 h of polymerization. Scale bar denotes 10 μm. Quantification of filament elongation rate normalized to the control (*n* = 3). Nucleation rate defined as the slope of number of filaments per frame quantified in 3–5 frames (with 30 s intervals) normalized to the control (*n* = 3). Statistical analysis was performed with one-way ANOVA and Bonferroni post hoc. n.s. = not significant in comparison to control (MBP). (**B**) Pyrene-polymerization assay. Actin filaments (5 μM, 20% pyrenyl-labeled) were polymerized and the increase in fluorescence of pyrene-actin during polymerization was monitored for 60 min. Representative curves are shown (**left**: raw background-corrected data, **right**: data normalized to endpoint). The buffer slightly slows down actin polymerization in comparison to the control with ddH_2_O, while there appears to be only marginal variation between the control with MBP (R = 0.5) and the buffer. (**C**) Actin networks (4 μM) were polymerized at RT for > 2 h and visualized with phalloidin-488. Images were taken in a confocal microscope (63×/1.4 NA objective, 10 μm z-stacks). Scale bar denotes 50 μm.

Recombinant DRR1 proteins were expressed and purified as maltose binding protein (MBP) fusion proteins in order to enhance stability and solubility. In our hands, various efforts to purify DRR1 without a tag [90] revealed insufficient stability of DRR1 [23]. We observed that with only a small tag this protein was prone to aggregation at high concentrations and required some urea (1M) and sodium dodecyl sulfate (SDS) (0.1%) to keep it in solution. Similarly, DRR1 turned out to be unstable when the MBP-tag was cleaved off. Therefore, control conditions with buffer only and with MBP only were included in all experiments with recombinant DRR1 proteins. Since there were no detectable differences in the results between buffer and MBP conditions (see Appendix Figure A5), the latter is shown in all figures as control. Proteins were expressed in *Escherichia coli* BL21(DE3)pLysS bacteria (Life Technologies) induced by 0.3 mM isopropyl-beta-D-1-thiogalactopyranoside (IPTG) for 2 h at 37 °C. The bacterial pellets were lysed by the freeze-thaw method in a dry-ice ethanol bath and then re-suspended in lysis buffer (binding buffer supplemented with protease inhibitor cocktail, 1 mg/mL lysozyme, 0.1 mM Phenylmethane sulfonyl fluoride (PMSF) and 1 mM Dithiothreitol (DTT)), incubated on ice for 1 h, and sonicated. The lysates were cleared by centrifugation at 48,400× *g* for 1 h at 4 °C (Beckmann Avanti J-25, Krefeld, Germany) and then filtered through a 0.22 µm syringe filter. The ÄKTA purifier system (General Electrics Healthcare) was used for protein purification with affinity chromatography (MBPTrap HP, 1 mL, GE Healthcare) and gel filtration (Superdex200 10/300 GL, GE Healthcare) as a second step. All buffers used were first filtered through a 0.22 µm filter and then degassed. Bacterial lysates were loaded on equilibrated MBPTrap columns after clearing and filtration at a flow rate of 0.5 mL/min with 15 mL binding buffer (20 mM Tris-HCl pH 7.4, 200 mM NaCl, 1 mM Ethylenediaminetetraacetic acid (EDTA), 1 mM DTT), washed with 5 mL binding buffer, and eluted with 10 mL elution buffer (20 mM Tris-HCl pH 7.4, 200 mM NaCl, 1 mM EDTA, 10 mM maltose, 1 mM DTT). Samples containing recombinant protein as controlled by SDS-PAGE and Coomassie staining were pooled and concentrated with Vivaspin 2, MWCO 30 kDa, columns (GE Healthcare). The buffer was changed to Superdex running buffer with (20 mM Tris-HCl pH 7.4, 150 mM NaCl, 1 mM DTT). A Superdex200 10/300 GL column (GE Healthcare) was used for gel filtration at a flow rate of 0.5 mL/min. Collected samples were loaded and analyzed with SDS-PAGE, with subsequent pooling of samples containing recombinant protein. Protein concentration was measured with UV absorbance at 280 nm and with colloidal Coomassie stained SDS-PAGE with a protein standard and densitometry.

Recombinant Profilin2a from mouse tagged with glutathione s-transferase (GST) was expressed from *E. coli* and purified with 2–4 mL glutathion sepharose 4B resin (GE Healthcare) in disposable columns. Binding and elution was performed in 50 mM Tris-HCl pH 7.0, 150 nM NaCl, 1 mM EDTA, 1 mM DTT. Elution of Profilin was performed by cleaving off the tag overnight at 4 °C with PreScission Protease (GE Healthcare). Protein concentration was determined by UV absorbance at 280 nm. Dialysis was performed against 20 mM Tris-HCl pH 7.0, 150 mM NaCl, 1 mM EGTA, 1 mM DTT.

All proteins were aliquoted and frozen in liquid nitrogen. Fresh aliquots of recombinant DRR1 or Profilin protein were used for all experiments.

### 4.5. Co-Immunoprecipitation

For co-immunoprecipitation (CoIP), HEK-293 cells transfected with plasmids expressing EGFP-fusion proteins were lysed with 200 µL ice-cold lysis buffer (10 mM Tris-HCl pH 7.5, 150 mM NaCl, 0.5 mM EDTA, 0.5% NP-40, 1:100 protease inhibitor cocktail P2714 from Sigma-Aldrich). The extract was incubated for 1 h on ice, diluted with 700 µL wash buffer (10 mM Tris-HCl pH 7.5, 150 mM NaCl, 0.5 mM EDTA, 1:100 protease inhibitor cocktail), and centrifuged for 10 min at 13,000 rpm to remove cell debris. Lysates were incubated with 25 µL GFP-Trap pre-equilibrated agarose beads (ChromoTek, Planegg-Martinsried, Germany) for 1 h at 4 °C. The beads were washed two times with 1 mL wash buffer and samples were eluted by incubation for 10 min at 95 °C in 50 µL 1× Laemmli sample buffer (1% SDS, 8% glycerol, 32 mM Tris-HCl pH 6.8, 5% mercaptoethanol, bromophenol blue). Fifteen microliters (15 μL) of each input/elution sample were loaded on gels for detection of Co-IP signals, and 5 μL were loaded for detection of EGFP-fusion-proteins. To calculate relative actin binding, IP and CoIP bands were revealed using an enhanced chemiluminiscence system (Millipore), detected with the Chemidoc system (BioRad, Munich, Germany), and quantified by densitometry (using the ImageLab 4.1 Software from Bio-Rad) with background signal, corresponding to areas of the membrane without signal, subtracted. Next, the corrected grey density value of the co-precipitated actin was referred to its corresponding value of the precipitated DRR1 protein and the actin/DRR1 ratio was defined as “actin binding”. To be able to compare the values between different experiments and blots, the “average actin binding” of all DRR1 proteins (wt, dN, dC, dM, and M) for each experiment was calculated and “actin binding” of each mutant was normalized to this average actin binding of the respective experiment. Therefore, “1.0” at the y-axis of Figure 1C denotes the (arbitrary value of) average actin binding of the DRR1 proteins and carries no further meaning.

### 4.6. Dual Luciferase Reporter Assay

For SRF reporter gene assays, Simian virus 40 promoter-driven non-secretory Gaussia luciferase expression vector [91] (10 ng per well in a 96-well-plate) was co-transfected in HEK-293 cells with SRF reporter plasmid (25 ng) to correct for transfection efficiency and the respective test plasmids (150 ng per well). The SRF reporter 3DA.luc and mDia1-dDAD plasmids were kind gifts from Robert Grosse (Universität Marburg, Germany). SRF activity was stimulated with 20% FBS or inhibited (0.5% FBS) for 16–18 h twenty-four hours after transfection. Cell lysis was performed with 50 µL passive lysis buffer (0.2% Triton X-100, 100 mM K_2_HPO_4_/KH_2_PO_4_ pH 7.8) for 30 min at room temperature. Activity of the firefly luciferase was measured in white microtiter plates in a luminometer ((TriStar LB941 Luminometer, Berthold Technologies, Bad Wildbad, Germany)) by adding 50 µL Firefly substrate solution (3 mM MgCl2, 2.4 mM ATP, 120 mM D-Luciferin) to 10 µL lysate. Then, 50 µL of Gaussia substrate solution (1.1 M NaCl, 2.2 mM Na_2_EDTA, 0.22 M K_2_HPO_4_/KH_2_PO_4_ pH 5.1, 0.44 mg/mL bovine serum albumin (BSA), Coelenterazine 3 µg/mL) was added to the same well to quench the firefly reaction and measure Gaussia luminescence with a 5 s delay. Firefly luminescence was corrected with Gaussia values to calculate Firefly activity data. The SRF activity in the serum-stimulated control with pRK5 was set to 1 in order to compare different experiments.

### 4.7. Chemical Crosslinking

HEK-293 cells were transfected with wt and mutant DRR1 expressing plasmids by electroporation. After cultivation for two days, cells were detached, washed, and then incubated in conjugation buffer (PBS with 1 mM EDTA) with either 200 μM crosslinker BMB (1,4-bismaleimidobutane, a crosslinker with a spacer arm length of 10.9 Å generating chemical bonds between sulfhydryl groups) or DMSO as control at 4 °C on a shaker for 2 h. Samples were quenched by incubation for 30 min at 4 °C in quenching buffer (10 mM DTT in PBS). Finally, protein extracts were prepared by centrifuging the cells and resuspension in SDS-lysis (20 mM Tris-HCl pH 7.4, 3.3% sucrose, 0.66% SDS, 1:100 protease inhibitor cocktail), short sonication and heating to 95 °C for 5 min. Protein concentration was determined with the bicinchoninic acid (BCA) method. A total of 5–10 μg protein were loaded on SDS-PAGE for Western blot analysis.

### 4.8. Subcellular Fractionation

HEK-293 cells were transfected with wt or mutant DRR1 expressing plasmids; after two days of cultivation, cells were trypsinized, washed with PBS, and resuspended in 250 μL hypotonic lysis buffer (10 mM HEPES pH 7.9, 10 mM KCl, 0.5 mM EDTA, 0.1% NP-40, 10% glycerol, 1 mM DTT, and 1:100 protease inhibitor cocktail (freshly added)) per 10 cm dish. After 10 min on ice and brief vortexing, disruption of the outer cell membrane was analyzed in the microscope. Centrifugation was at 6500 rpm for 30 s at 4 °C), followed by transfer of the supernatant (containing the cytosolic proteins) to a fresh tube. The pellet was washed three times with 500 μL hypotonic lysis buffer and the nuclei were lysed by incubating in 200 μL SDS-lysis buffer (1× diluted from 3× which is: 62.5 mM Tris-HCl pH 7.4, 10% sucrose, 2% SDS, 1:100 protease inhibitor cocktail (freshly added)) 5 min at 95 °C and short sonication. After centrifugation, the supernatant was transferred to a fresh tube. Protein concentration of cytosolic and nuclear fractions was determined by BCA and the samples were analyzed by SDS-PAGE and Western blot. About 7–10 μg cytosolic fraction and the same volume of the corresponding nuclear fraction were loaded.

### 4.9. HeLa Cell Spreading

HeLa cells were transfected with DRR1 in pEGFP (using TurboFect), harvested on the next day and replated on fibronectin-coated (50 µg/mL) 12 mm round coverslips in 24-well plates (Merck Millipore, Darmstadt, Germany). Cell spreading was stopped after 30 min of spreading at 37 °C by fixing cells with 4% paraformaldehyde for 20 min at room temperature. The actin cytoskeleton was stained with Alexa Fluore 594-phalloidin and stained cells were placed on glass slides with a drop of Prolong Gold Antifade Medium (Life Technologies). A laser scanning microscope was used for analysis (10×/0.40 NA or 40×/1.15 NA objective, LSM FV-1000, Olympus, Shinjuku, Japan). Up to 10 fields were randomly selected with 50–100 cells each and analyzed with ImageJ software. The images were scaled, the phalloidin channel was thresholded (lower threshold level 250, upper threshold level 4095 for a 16-bit image) and adjacent cells were separated by the “Watershed” algorithm (controlling manually for correct cell separation). The thresholded phalloidin channel was used determine cell size in the original phallidin channel and to measure the mean gray value per cell in the EGFP channel using the “Analyze Particles” algorithm. Cells with a mean gray value > 500 in the EGFP channel were determined to be transfected. Cells with a mean gray value of > 500 were defined as transfected. In order to compare different conditions, the mean area of transfected cell was normalized with the mean area of untransfected cells in the same condition.

### 4.10. Fluorescence Recovery after Photobleaching (FRAP)

FRAP was analyzed in HeLa cells plated on 50 µg/mL fibronectin-coated 35 mm glass dishes transfected with TurboFect. Per glass dish, 1 µg GFP-actin was cotransfected with 3 µg (unlabeled) DRR1 constructs in pRK5. After 24 h, the medium was changed to fresh medium. Time-lapse image frames with 2 s intervals for 5 min were acquired in the confocal microscope (20×/0.8 NA objective, 5× zoom, C.A. 200 µm, LSM FV-1000, 2% laser power). Prior to bleaching, five frames were recorded. At an image resolution of 320 × 320 px, bleaching was performed with the circular “TurboTool” for 1000 ms at 100% 488-laser power. This led to a bleach of the GFP-fluorescence of about 80%. ImageJ software was used to quantify FRAP. First, the mean gray value measured in the bleached area was divided through an equally-sized arbitrary non-bleached area within the same cell in each frame to correct for acquisition photobleaching and possible laser fluctuations. In order to normalize each fluorescence recovery curve to compare different cells and conditions, the mean gray value of the bleached area immediately after bleaching (C(t0)) was set to 0, while the pre-bleached value (C(pre)) was set to one. The mean gray value of each time point was, thus, normalized using the following formula described in [92]: N(t) = [C(t) − C(t0)]/[C(pre) − C(t0)]. The average gray values in the time lapse imaging was finally averaged across different cells and experiments and plotted as depicted. “N” refers to the number of cells analyzed from 2–3 independent experiments.

### 4.11. Cellular Stainings

HeLa cells were seeded on 50 µg/mL fibronectin-coated glass coverslips (Merck Millipore), placed in 24-well-plates and transfected using TurboFect as described above. For immunofluorescence, cells were fixed 24 h after transfection with 4% paraformaldehyde in PBS for 20 min at room temperature. Cells were permeabilized with 0.1% Triton X-100 for 10 min and blocked with 10% goat serum for 1 h (both in PBS at room temperature). Primary antibodies were diluted 1:200 in 0.1% Triton X-100/PBS and incubated overnight at 4 °C. Secondary antibodies were diluted 1:500 in 0.1% Triton X-100/PBS and incubated for 3 h at room temperature. The following antibodies were used: rabbit-anti-DRR1 (Biogenes, Berlin, Germany), Alexa Fluor 647-goat-anti-rabbit (Life Technologies). For staining of actin filaments, cells were incubated with 165 nM AlexaFluor 594, or 546 phalloidin (Life Technologies) in PBS for 20 min at room temperature. Nuclei were counterstained with 1 µg/mL DAPI in PBS for 15 min at room temperature.

### 4.12. Colocalization Analysis

Images for colocalization analysis were taken with the 40×/1.15 NA objective, 3× zoom, and a pinhole of 200 μm. Colocalization analysis was performed in ImageJ with the plugin “Coloc 2” using individual cells as ROI and a point spread function of 4.25. Pearson’s correlation coefficient (PCC) R and Costes *p* value were calculated using 100 Costes randomizations. For each condition, 5–15 randomly-selected cells from two independent experiments were analyzed.

### 4.13. Quantification of Mean Cellular F-actin Content

Quantification of mean cellular F-actin content was performed in ImageJ. Shortly, the images were scaled, the phalloidin channel was thresholded (lower threshold level 150, upper threshold level 4095) and adjacent cells were separated by the “Watershed” algorithm. Correct cell separation was double-checked manually. By means of the thresholded phalloidin channel, the mean grey value of individual cells in both original channels was measured. Cells with a mean gray value of >500 were defined as transfected. For each condition, the mean gray value of F-actin in transfected cells was normalized to the untransfected cells in order to compare different samples.

### 4.14. Actin Preparation

G-actin was obtained from rabbit skeletal muscle actin and labeled with pyrene, as described previously [67,93]. All in vitro actin experiments except the F-actin co-sedimentation were performed in the lab of Andreas R. Bausch. In all experiments, “R” refers to the molar ratio of recombinant protein and actin. Experiments were performed under reducing conditions with 1 mM DTT.

### 4.15. Pyrene-Actin Polymerization Assay

Actin polymerization was monitored by the increase in fluorescence of 20% pyrenyl-actin at 407 nm (excitation at 365 nm) in a fluorescence spectrometer (Jasco FP-8500, Gross-Umstadt, Germany). The final concentration of actin in the reaction was 4 µM. DRR1 proteins were added to G-actin in a constant volume and polymerization was induced by the addition of 1:10 volume of 10× F-buffer (250 mM Tris-HCl pH 7.4, 250 mM KCl, 40 mM MgCl_2_, 10 mM EGTA, 10 mM ATP, 10 mM DTT). The polymerization was monitored for 1 h at 21 °C with a cycle interval of 5.5 s.

### 4.16. Actin-Filament Elongation and Nucleation Assay

For visualization of single filament polymerization samples containing 1× F-buffer and recombinant proteins (in a constant volume) were prepared. Polymerization was induced by the addition of G-actin (0.5 or 1 µM final concentration). The sample was then immediately pipetted into a flow chamber consisting of two high precision coverslips (60 × 24 mm and 20 × 20 mm, Carl Roth, Karlsruhe, Germany) separated by vacuum grease and placed in a TIRF or confocal microscope (TIRF: Leica DMI6000B, 100×/1.47 NA oil immersion objective, confocal: 63×/1.4 NA oil immersion objective, 5× optical zoom, Leica TSC SP5, Solms, Germany). Samples prepared with 10% actin-ATTO488 were visualized in a TIRF microscope, samples with Alexa Fluor 488-phalloidin were visualized in the confocal microscope.

To avoid unspecific surface interactions casein was added to the samples in 0.15 mg/mL. The larger coverslips were previously cleaned with a plasma cleaner (40–50 s at 4–6 mbar) and N-ethylmaleimide-modified heavy meromyosin (NEM-HMM, 2.7 µg/mL diluted in F-buffer) was bound to the surface to keep actin filaments close to the surface during live visualization. The chambers were washed with 1× F-buffer prior to applying the sample. The time between the addition of actin to the sample and initiation of the visualization was 2 min. Time-lapse images of polymerization were acquired for 10 min every 3 s.

The image analysis for single filament elongation rate was performed with ImageJ. In all images the background was subtracted and, subsequently, brightness and contrast were adjusted if necessary for ideal visualization. Shortly thereafter, a segmented line was drawn along the filament and plotted time versus filament length (i.e., fluorescence intensity) with the plugin “multiple kymograph”. The slope of this linear graph corresponds to the filament elongation rate at the barbed end and was as follows for the MBP control: about 5.6 actin monomers per second in the TIRF assay (0.5 µM actin), and 11.5 actin monomers per second in the confocal assay (1 µM actin), based on the published value of 0.0027 µm/actin monomer in actin filaments [94]. The filament elongation rate of the MBP control was set to 100% in order to compare different experiments. The polymerization speed strongly depended on the actin preparation. This is in accordance to values described in the literature for ATP-actin at similar buffer conditions [95]. Barbed and pointed ends could be easily distinguished in the ImageJ graph in the control, as the elongation rate is much lower at the pointed end. For samples containing DRR1 this was not possible due to the strong inhibition of elongation. Thus, profilin (R = 3) was added to the samples to block pointed end elongation. Ten filaments from three independent experiments were measured for each condition.

For nucleation analysis (both in TIRF and confocal assays), filaments in 4–8 frames with 30 s intervals were counted manually from 3–6 independent experiments, respectively. The number of filaments was plotted versus time of polymerization and the slope of the resulting linear graph was defined as relative nucleation rate. The MBP control was set to 1. MBP exerted no significant effect on polymerization or nucleation in comparison to buffer conditions (see Appendix Figure A5).

### 4.17. Reconstituted Actin Networks

Samples with 4 µM actin and 2 µM recombinant DRR1 proteins (R = 0.5, wt also in R = 0.1 and 0.25) at a constant volume were prepared for in vitro actin networks and Alexa Fluor 488-phalloidin was added to visualized actin (0.08 µM, Life Technologies). Furthermore, casein was added to the samples in 0.15 mg/mL to avoid unspecific surface interactions. After induction of polymerization by addition of 1:10 volume of 10× F-buffer, the samples were placed into a flow chamber. The chamber was sealed with vacuum grease and samples were polymerized at room temperature for 1.5–2 h protected from light. At equilibrium of polymerization, networks were visualized using a confocal microscope (63×/1.4 NA oil immersion objective, 1× or 3× optical zoom, Leica TSC SP5). Z-stacks of each sample were taken with a 10 µm depth and slices with 0.38 µm step size and maximum projections of the staces were generated with ImageJ software. In addition, the background of this maximum projections was subtracted and brightness and contrast were automatically adjusted.

### 4.18. F-Actin Co-Sedimentation Assay

Polymerization of 1 µM G-Actin was induced by the addition of 1:10 volume of 10× F-buffer for 1 h at room temperature. Freshly thawed recombinant DRR1 proteins were added to the F-actin samples at 0.5 µM final concentration in a constant volume and incubation on ice was performed for 30 min to allow proteins to bind to F-actin. The samples were centrifuged at 150.000× *g* for 1 h at 21 °C (Beckmann LB-70M). An aliquot was taken prior to centrifugation (representing “T” = total protein) and after centrifugation supernatant and pellet samples were collected (“S” = supernatant, “P” = pellet). Supernatant samples contain G-actin and non-sedimented protein, while F-actin and F-actin-binding proteins are found in the pellet. All samples were loaded on SDS-PAGE, stained with colloidal Coomassie and subsequently analyzed by densitometry scanning (ChemiDoc Imaging System, Bio-Rad Laboratories, Munich, Germany). Since the pellet fractions are easily contaminated by residual supernatant proteins, the fraction of co-sedimented protein was calculated by the subtraction of the amount in the supernatant fraction from the amount of total protein determined on the same gel as the more reliable method. To determine the background precipitation of each protein, the proteins were each centrifuged alone (i.e., without the presence of F-actin) as a control. The amount of pelleted protein in these samples (^−Actin^) was subtracted from the co-sedimented protein amount in the samples with F-actin (^+Actin^). This value was then related to (i.e., divided by) the total and is presented as % in Figure 1E. Thus, the amount of DRR1 (wt and mutants) and MBP control co-sedimenting with F-actin was determined by ([(T^+Actin^ − S^+Actin^) − (T^−Actin^ − S^−Actin^)]/T^+Actin^) × 100. This method was highly reproducible and consistent.

Control experiments were performed with ddH_2_O, buffer and MBP at R = 0.5, respectively. No differences were detected in the samples containing MBP vs. buffer and MBP controls are shown in all panels. Both buffer and MBP sample, however, seemed to slightly slow down actin polymerization in comparison to ddH_2_O samples (see Appendix A, Figure A5).

### 4.19. Statistical Analysis

The commercially available program SigmaPlot 14.0 (Erkrath, Germany) was used for statistical analysis. A one-way ANOVA (analysis of variants) was performed followed by Bonferroni *post hoc* analysis for multi-group comparisons. The level of significance was set at *p* < 0.05. All data are presented as mean ± SEM. Grubb’s test (with alpha = 0.05) was run to identify significant outliers.

## 5. Conclusions

This study characterizes the tumor suppressor and stress-regulated protein DRR1 as actin binding protein that affects several aspects of actin dynamics such as nucleation, elongation, capping and bundling of F-Actin. We are only beginning to understand the cellular and physiological implications. Through induction of DRR1 stress changes the cellular make-up of actin dynamic regulators. The interplay of DRR1 with the array of other actin binding proteins needs further exploration, as well as the cellular effects that are expected to extend beyond G-actin dependent transcription and cell spreading. Future experiments should also refine the analysis of the structural and functional changes of neuronal networks that might contribute to stress-related psychiatric diseases.

## Figures and Tables

**Table 1:** Schematic overview of DRR1’s molecular effects on actin dynamics.

	F-Actin Binding	Bundling	Filament Elongation	Nucleation	Capping
DRR1 wt	+ + +	+ + +	− − −	+ +	+ + +
dN	+	+ +	0	0	0
dC	+ +	+	− (n.s.)	+ (n.s.)	+
dM	+ +	+	− − −	+ (n.s.)	+ + +
M	0	0	0	0	0

Summary of the data presented in Figure 1, Figure 2, Figure 4 and Figure 5. + enhancement, − decrease, 0 no effect, n.s. not significant.

**Table 2:** Schematic overview of DRR1 effects on actin-dependent cellular processes.

DRR1	Colocalization F-Actin	Cellular F-Actin	Actin Treadmillling	Cell Spreading	SRF Activation
wt	+++	+++	− − −	− − −	+ + +
dN	++	+	− − −	−	+ +
dC	+	0	0	− − −	0
dM	++	++	0	−	+
M	0	0	0	0	0

Summary of the data presented in Figure 3 and Figure 6. + enhancement, − decrease, 0 no effect.

## Abbreviations

ANOVA	Analysis of variants
ATP	Adenosine triphosphate
BCA	Bicinchoninic acid
BMB	1,4-Bismaleimidobutane
BSA	Bovine serum albumin
CA3	Cornu Ammonis region 3 (hippocampal region)
CaMKIIb	Ca2+/calmodulin-dependent protein kinase IIb
CMV	Cytomegalovirus
CoIP	Co-immunoprecipitation
DRR1	Down-regulated in renal cell carcinoma 1 (also known as FAM107A or TU3A)
DTT	Dithiothreitol
EDTA	Ethylenediaminetetraacetic acid
EGFP	Enhanced green fluorescent protein
F-actin	Filamentous actin
FAM107A	Family with sequence similarity 107, member A
G-actin	Globular actin
GFP	Green fluorescent protein
HEK-293	Human embryonic kidney 293 cells
MBP	Maltose binding protein
PCC	Pearson’s correlation coefficient
PMSF	Phenylmethane sulfonyl fluoride
R	Molar ratio DRR1 (wt or mutants):actin
RT	Room temperature
SDS	Sodium dodecyl sulfate
SDS-PAGE	Sodium dodecyl sulfate polyacrylamide gel electrophoresis
SRF	Serum response factor
TIRF	Total internal reflection fluorescence
TU3A	Tohoku University cDNA clone A on chromosome 3
wt	Wild-type
